# Supplementary material for: CAR-NK Cells: A Chimeric Hope or a Promising Therapy?
Source: Cancers (Basel). 2022 Aug 8;14(15):3839. doi: 10.3390/cancers14153839 (PMC9367380; doi:10.3390/cancers14153839)
Supplement: Supplementary file 1 [file cancers-14-03839-s001.zip › cancers-1823101-supplementary.pdf]

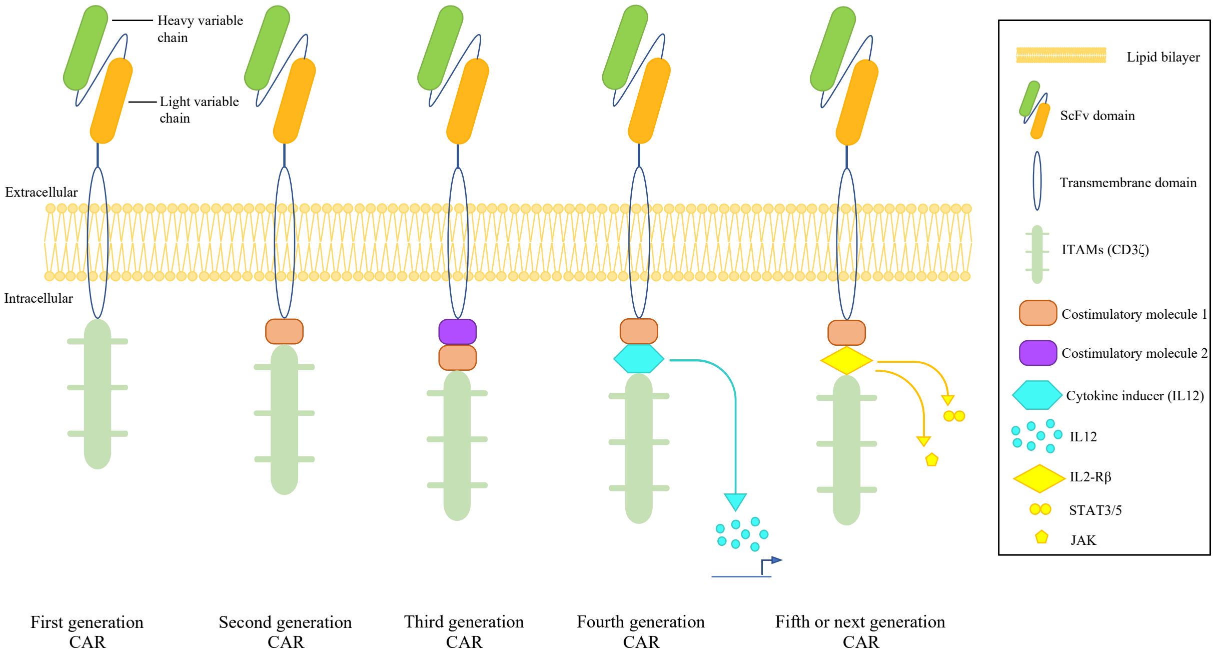

Supplementary Figure S1: Different generations of chimeric antigen receptors. All CAR generations are composed of the same extracellular domain which is a single chain variable fragment (a heavy variable chain and a light variable chain). In the intracellular domain, first generation CAR is composed of only a CD3ξ (immunoreceptor tyrosine based activation motifs, ITAM). Second generation CARs have, in addition to their CD3ξ, a co-stimulatory molecule (usually CD28 or 4-1BB). Third generation CARs include a second co-stimulatory domain. Fourth generation CAR has one costimulatory molecule and their ITAM domain, in addition to a constitutively or inducibly expressed cytokine (IL-12) upon activation, in order to enhance their cytotoxicity. Fifth or next generation CARs also have one costimulatory molecule and ITAM domains, paired with a truncated intracellular domain of cytokine receptors (IL-2Rβ chain fragment with a STAT3/5 binding motif).

Supplementary Table S1: CAR-NK trials on clinicaltrials.gov:

TITLE	CONDITIONS	PHASE	INTERVENTION	LOCATIONS	ENROLLEMENT	NCT NUMBER	STATUS
**Anti-CD19 CAR NK Cell Therapy for R/R Non-Hodgkin Lymphoma**	relapsed/refractory B NHL	Early Phase 1	**Anti-CD19 CAR-NK Cells**	Xinqiao Hospital, Departement of hematology, Chongqing, China	9 patients	NCT04639739	Not recruiting
**Study of Anti-CD22 CAR NK Cells in Relapsed and Refractory B Cell Lymphoma**	relapsed/refractory B NHL	Early Phase 1	**Anti-CD22 CAR-NK Cells**	USA	9 patients	NCT03692767	Not recruiting
**Study of Anti-CD19 CAR NK Cells in Relapsed and Refractory B Cell Lymphoma**	relapsed/refractory B NHL	Early Phase 1	**Anti-CD19 CAR-NK Cells**	USA	9 patients	NCT03690310	Not recruiting
**A Phase I/II Study of Universal Off-the-shelf NKG2D-ACE2 CAR-NK Cells for Therapy of COVID-19**	COVID-19	Phase 1/2	**NK cells, IL15-NK cells, NKG2D-ACE2 CAR-NK Cells**	Chongqing Public Health Medical Center, Chongqing, China	90 patients	NCT04324996	Recruting
**Study of Anti-Mesothelin Car NK Cells in Epithelial Ovarian Cancer**	Epithelial Ovarian Cancer	Early Phase 1	**Anti-Mesothelin Car-NK Cells**	USA	30 patients	NCT03692637	Not recruiting
**Study of Anti-PSMA CAR NK Cell in Castration-Resistant Prostate Cancer**	Castration-resistant prostate cancer	Early Phase 1	**Anti-PSMA CAR-NK cells**	USA	9 patients	NCT03692663	Not recruiting
**Pilot Study of NKG2D-Ligand Targeted CAR-NK Cells in Patients With Metastatic Solid Tumours**	Metastatic solid tumors	Phase 1	**NKG2D-ligand CAR-NK Cells**	Third Affiliated Hospital of Guangzhou Medical University, Guangzhou, China	30 patients	NCT03415100	unkown
**Clinical Research of ROBO1 Specific CAR-NK Cells on Patients With Solid Tumors**	Solid tumors	Phase 1/2	**ROBO1 CAR-NK cells**	Suzhou Cancer Center, Nanjing Medical University, Radiation therapy department, Suzhou, [illegible]	20 patients	NCT03940820	Recruting
**Clinical Research of Adoptive BCMA CAR-NK Cells on Relapse/Refractory MM**	relapsed/refractory multiple myeloma	Phase 1/2	**BCMA CAR-NK92 cells**	Wuxi People's Hospital, Nanjing Medical University, Hematology department, Wuxi, [illegible]	20 patients	NCT03940833	Recruting
**Study of Anti-CD19/CD22 CAR NK Cells in Relapsed and Refractory B Cell Lymphoma**	relapsed/refractory B NHL	Early Phase 1	**Anti-CD19/CD22 CAR-NK Cells**	Beijing Cancer Hospital, China	10 patients	NCT03824964	unkown
**CAR-pNK Cell Immunotherapy for Relapsed/Refractory CD33+ AML**	relapsed/refractory AML	Phase 1/2	**Anti-CD33 CAR-NK Cells**	PersonGen BioTherapeutics, Suzhou, Jiangsu, China	10 patients	NCT02944162	unkown
**PCAR-119 Bridge Immunotherapy Prior to Stem Cell Transplant in Treating Patients With CD19 Positive Leukemia and Lymphoma**	CD19+ B cell malignancies (ALL, CLL, FL, MCL, DLBCL, B cell prolymphocytic leukemia) prior to HSCT	Phase 1/2	**Anti-CD19 CAR-NK Cells**	PersonGen BioTherapeutics, Suzhou, Jiangsu, China	10 patients	NCT02892695	unkown
**Safety of Intravenous Allogeneic Engineered Natural Killer Cells in Adults With AML or MDS**	relapsed/refractory AML and MDS	Phase 1	**NKX101 CAR-NK Cells**	Colorado Blood Cancer Institute, Denver ; Winship Cancer Institute, Atlanta ; Taussig Cancer Institute, Cleveland ; Sarah Cannon at TriStar Bone Marrow Transplant Center, Nashville ; USA	90 patients	NCT04623944	Recruting
**Clinical Research of ROBO1 Specific BiCAR-NK Cells on Patients With Pancreatic Cancer**	Pancreatic Cancer	Phase 1/2	**BiCAR-NK Cells (ROBO1 CAR-NK cells)**	Shanghai Ruijin Hospital, Departement of radiology, Shanghai, China	9 patients	NCT03941457	Recruting
**Clinical Research of ROBO1 Specific BiCAR-NK/T Cells on Patients With Malignant Tumor**	Malignant Tumor	Phase 1/2	**BiCAR-NK/T Cells (ROBO1 CAR-NK/T cells**	Suzhou Kowloon Hospital, Hematology departement, Suzhou, Jiangsu, China	20 patients	NCT03931720	Recruting
**CAR.CD19-CD28-zeta-2A-iCasp9-IL15-Transduced Cord Blood NK Cells, High-Dose Chemotherapy, and Stem Cell Transplant in Treating Participants With B-cell Lymphoma**	CD19 positive recurrent or refractory NHL: MCL, DLBCL, FL	Phase 1/2	BEAM-ASCT, Rituximab, **umbilical cord blood derived CAR NK cells**	M D Anderson Cancer Center, Houston, Texas, USA	0 patients	NCT03579927	Withdrawn
**Umbilical & Cord Blood (CB) Derived CAR-Engineered NK Cells for B Lymphoid Malignancies**	CD19+ B malignancies (ALL, CLL, NHL)	Phase 1/2	**iC9/CAR.19/IL15-Transduced CB-NK Cells**	University of Texas MD Anderson Cancer Center, Houston, Texas, USA	36 patients	NCT03056339	Recruting
**KG2D CAR-NK Cell Therapy in Patients With Relapsed or Refractory Acute Myeloid Leukemia**	relapsed/refractory AML	phase 1	**NKG2D CAR-NK cells**	Hebei Yanda Lu Daopei Hospital, Sanhe, Hebei, China	9 patients	NCT05247957	Recruting
**Clinical Study of HLA Haploidentical CAR-NK Cells Targeting CD19 in the Treatment of Refractory/ Relapsed B-cell NHL**	relapsed/refractory B NHL	phase 1	**Anti-CD19 CAR-NK Cells**	2nd Affiliated Hospital, School of Medicine, Zhejiang University, Hangzhou, Zhejiang, China	25 patients	NCT04887012	Recruting
**NKG2D CAR-NK Cell Therapy in Patients With Refractory Metastatic Colorectal Cancer**	refractory metastatic colorectal cancer	Phase 1	**NKG2D CAR-NK cells**	The First Affiliated Hospital, Zhejiang University, Hangzhou, Zhejiang, China	38 patients	NCT05213195	Recruting
**Study of Anti-CD33/CLL1 CAR- NK in Acute Myeloid Leukemia**	AML	Early Phase 1	**Anti-CD33/CLL1 CAR- NK Cells**	Wuxi People's Hospital, Wuxi, Jiangsu, China	18 patients	NCT05215015	Recruting
**Study of Anti-5T4 CAR-NK Cell Therapy in Advanced Solid Tumors**	advanced solid tumors	Early Phase 1	**anti-5T4 CAR-NK cells**	Wuxi People's Hospital, Wuxi, Jiangsu, China	40 patients	NCT05194709	Recruting
**Anti-CD33 CAR NK Cells in the Treatment of Relapsed/ Refractory Acute Myeloid Leukemia**	AML	Phase 1	**Anti-CD33 CAR-NK Cells**	Department of Hematology, Xinqiao Hospital, Chongqing, Chongqing, China	27 patients	NCT05008575	Recruting
**Anti-BCMA CAR-NK Cell Therapy for the Relapsed or Refractory Multiple Myeloma**	refractory multiple myeloma	Early Phase 1	**anti-BCMA CAR-NK cells**	Department of Hematology, Xinqiao Hospital, Chongqing, Chongqing, China	27 patients	NCT05008536	Recruting
**Immunotherapy Combination: Irradiated PD-L1 CAR-NK Cells Plus Pembrolizumab Plus N-803 for Subjects With Recurrent/ Metastatic Gastric or Head and Neck Cancer**	Gastroesophageal junction cancers and advanced head and neck squamous cell cancer	phase 2	**Irradiated PD-L1 CAR-NK Cells**, Pembrolizumab, N-803 (IL15 superagonist)	National Institutes of Health Clinical Center, Bethesda, Maryland, United States	55 patients	NCT04847466	Recruting
**Study of Anti-5T4 CAR- raNK Cell Therapy in Locally Advanced or Metastatic Solid Tumors**	Locally Advanced or Metastatic Solid Tumors	Early Phase 1	**Anti-5T4 CAR-raNK Cells**	Shanghai East Hospital, Shanghai, Shanghai, China	136 patients	NCT05137275	Recruting
**CAR-pNK Cell Immunotherapy in MUC1 Positive Relapsed or Refractory Solid Tumor**	Hepatocellular Carcinoma, Non-small Cell Lung Cancer, Pancreatic Carcinoma, Triple-Negative Invasive Breast Carcinoma, Malignant Glioma of Brain, Colorectal Carcinoma, Gastric Carcinoma	Phase 1/2	**anti- MUC1 CAR-pNK cells**	PersonGen BioTherapeutics (Suzhou) Co., Ltd., Suzhou, Jiangsu, China	10 patients	NCT02839954	unkown
**CAR-pNK Cell Immunotherapy in CD7 Positive Leukemia and Lymphoma**	CD7+ AML, precursor T cell lymphoblastic leukemia/lymphoma, T-cell prolymphocytic leukemia, T-cell LGL leukemia, Peripheral T cell lymphoma NOS, angioimmunoblastic T cell lymphoma, Extranodal NK/T-cell lymphoma nasal type, enteropathy type intestinal T cell lymphoma, hepatosplenic T cell lymphoma	Phase 1/2	**anti-CD7 CAR-pNK cells**	PersonGen BioTherapeutics (Suzhou) Co., Ltd., Suzhou, Jiangsu, China	10 patients	NCT02742727	Recruting
**Phase I/II Study of CD5 CAR Engineered IL15-Transduced Cord Blood-Derived NK Cells in Conjunction With Lymphodepleting Chemotherapy for the Management of Relapsed/Refractory Hematological Malignances**	hematological malignancies	Phase 1/2	**CAR.5/IL15- transduced CB derived anti CD5 CAR-NK cells**	M D Anderson Cancer Center, Houston, Texas, United States	48 patients	NCT05110742	Not recruiting
**phase I/II Study of CAR.70- Engineered IL15-transduced Cord Blood-derived NK Cells in Conjunction With Lymphodepleting Chemotherapy for the Management of Relapse/ Refractory Hematological Malignances**	B-Cell NHL, MDS, AML	Phase 1/2	**CAR.70/IL15- transduced CB-NK cells**	M D Anderson Cancer Center, Houston, Texas, United States	94patients	NCT05092451	Not recruiting
**FT576 in Subjects With Multiple Myeloma**	multiple myeloma	Phase 1	**FT576 (Allogenic CAR NK cells with BCMA expression)**	University of Alabama at Birmingham, Alabama; Colorado Blood Cancer Institute, Colorado; Indiana University Melvin and Bern Simon Comprehensive Cancer Center, Indiana; Tennessee Oncology, Tennessee; Froedtert Hospital, Medical College, Wisconsin, USA	168 patients	NCT05182073	Recruting
**Universal Chimeric Antigen Receptor-modified AT19 Cells for CD19+ Relapsed/Refractory Hematological Malignancies**	ALL, CLL, B cell NHL	Phase 1	**AT19 CAR-NK-CD19**	Union Hospital, Huazhong University of Science and Technology, Wuhan, Hubei, China	27 patients	NCT04796688	Recruting
**Cord Blood Derived Anti-CD19 CAR-Engineered NK Cells for B Lymphoid Malignancies**	B ALL, CLL, B NHL	Phase 1	Cord Blood derived CAR-NK-CD19	Union Hospital, Huazhong University of Science and Technology, Wuhan, Hubei, China	27 patients	NCT04796675	Recruting
**NKX019, Intravenous Allogeneic Chimeric Antigen Receptor Natural Killer Cells (CAR NK), in Adults With B-cell Cancers**	NHL, B ALL, Large B cell lymphoma, MCL, Indolent lymphoma, Waldenstrom macroglobulinemia, CLL, small lymphocytic lymphoma, agressive lymphoma	Phase 1	NKX019: allogenic anti CD19 Peripheral blood derived CAR NK cell expressing IL15	•Colorado Blood Cancer Institute, Colorado; University of Chicago, Illinois; The Cleveland Clinic Foundation, Ohio, United States •Institute of Haematology, Camperdown; St. Vincent's Hospital, Sydney, New South Wales,Royal Brisbane and Woman's Hospital, Queensland;Peter MacCallum Cancer Center [illegible]	60 patients	NCT05020678	Recruting
**A Study of CNTY-101 in Participants With CD19-Positive B-Cell Malignancies**	CD19 positive B cell malignancies	Phase 1	CNTY-101: allogeneic, iPSC-derived CAR-iNK cell therapy that has been engineered to express CD19 CAR, soluble IL-15, and an EGFR safety switch	USA	75 patients	NCT05336409	Not recruiting

LNH: non hodgekin lymphoma; AML: acute myeloid lymphoma; ALL: acute lymphoblastic leukemia; MDS: myelodysplasic syndrome; CLL : chronic lymphoid leukemia; MCL: mantle cell lymphoma; DLBCL: diffuse large B cell lymphoma; FL: Follicular lymphoma; BEAM: Carmustine, Etoposide, Cytarabine, Melphalan; ASCT: Autologuous hematopoietic stem cell transplantation; HSCT: hematopoietic stem cell transplantation

Supplementary Table S2: Key Points

1	NK cells represents about 5-15 % of circulating lymphocytes.	
2	Their cytotoxicity is antigen independent and doesn't need priming. It depend on a balance between inhibitory and activating signals from a complex interaction between their receptors and the ligands present on the target.	
3	Advantages over CAR-T cells	They don't cause GVHD and can be used in an allogenic setting, representing a huge benefit over autologous cells, supporting banking with a rapid availability of the product
		Superior quality and homogeneity of effectors cells
		Allogenic CAR-NK seem to have a better safety profile with less toxicity (few to no CRS and neurotoxicity).
		mechanisms of actions: classical cytotoxicity via the CAR and ADCC with the possibility to target a second tumoral antigen and limiting the risk of relapsed by antigen loss.
		Many sources to generate CAR-NK: PBMC, UCB units, NK92 cell line, iPSC.
		Possible generation of multiple batches from a single cellular source with a reduction in costs and the possibility for multiple injections (remains to be confirmed, according to the dose defined for a therapeutic benefit).
4	Disadvantages	NK cells derived from immatures cells (UBC, iPSC) have a $CD56^{bright}$ KIR^{neg} NKG2A+ CD16low profile making them very proliferative but less cytotoxic. Nevertheless, this should be overpassed by culture in presence of cytokines and transduction.
		PBMC derived NK cells have a more cytotoxic profile with CD56dim, CD16+, KIR+ but are less proliferative
		There remain significant problems in the production of CAR-NK cells. The optimal protocol for the introduction of foreign genetic material and subsequent expansion of the NK cells remains to be defined, making the development of feasible and reproducible GMP protocols still a challenge. The viability of the CAR-NK cells is central to the success of the therapeutic product, as the long-term persistence of tumor-specific CAR cells in the host is thought to promote the therapeutic efficacy.
		The interaction between NK inhibitory receptors (KIR and NKG2A) and their ligands (HLA-C, HLA-Bw4 and HLA-E) inhibits NK functions. To surpass this inhibition, further modifications are needed: selection of donors based on their HLA (especially related haploidentical donors) or by added cytokinic signals like IL15 or IL2.
		Like all allogenic HLA incompatible cells, the risk is immune rejection and the short persistence of the injected cells. Repeated injections and the use of cytokinic signals is a promising way to surpass this obstacle.

Supplementary Table S3: Abbreviations

**NK**	**Natural Killer**
MHC	Major Histocompatibility Complex
TRAIL	Tumor necrosis factor Related Apoptosis Inducing Ligand
HLA	Human Leukocyte Antigen
GVHD	Graft Versus Host Disease
GVL	Graft Versus Leukemia/lymphoma
NCR	natural cytotoxicity receptors
KIR	Killer cell Ig-like Receptors
ADCC	Antibody Dependent Cellular Cytotoxicity
Ig	Immunoglobulin
B-ALL	B Acute Lymphoblastic Leukemia
CAR	Chimeric Antigen Receptor
CRS	Cytokine Release Syndrome
TCR	T Cell Receptor
scFv	single chain variable Fragment
iPSCs	induced Pluripotent Stem Cells.
